# Second Case of HOIP Deficiency Expands Clinical Features and Defines Inflammatory Transcriptome Regulated by LUBAC

**DOI:** 10.3389/fimmu.2019.00479

**Published:** 2019-03-18

**Authors:** Hirotsugu Oda, David B. Beck, Hye Sun Kuehn, Natalia Sampaio Moura, Patrycja Hoffmann, Maria Ibarra, Jennifer Stoddard, Wanxia Li Tsai, Gustavo Gutierrez-Cruz, Massimo Gadina, Sergio D. Rosenzweig, Daniel L. Kastner, Luigi D. Notarangelo, Ivona Aksentijevich

**Affiliations:** ^1^Inflammatory Disease Section, National Human Genome Research Institute (NHGRI), NIH, Bethesda, MD, United States; ^2^Department of Laboratory Medicine, NIH Clinical Center (CC), Bethesda, MD, United States; ^3^Division of Pediatric Rheumatology, Children's Mercy Hospital, Kansas City, MO, United States; ^4^Office of Science and Technology, National Institute of Arthritis and Musculoskeletal and Skin Diseases (NIAMS), NIH, Bethesda, MD, United States; ^5^Laboratory of Clinical Immunology and Microbiology, National Institute of Allergy and Infectious Diseases (NIAID), NIH, Bethesda, MD, United States

**Keywords:** LUBAC, HOIP, HOIL1, SHARPIN, primary immunodeficiency, autoinflammation, CVID

## Abstract

**Background:** HOIP is the catalytic subunit of the linear ubiquitination chain assembly complex (LUBAC) that is essential for NF-κB signaling and thus proper innate and adaptive immunity. To date only one patient with HOIP deficiency has been reported with clinical characteristics that include autoinflammation, immunodeficiency, amylopectinosis, and systemic lymphangiectasia.

**Case:** We sought to identify a genetic cause of a disease for an 8 year-old girl who presented with early-onset immune deficiency and autoinflammation.

**Methods:** Targeted next generation sequencing of 352 immune-related genes was performed. Functional studies included transcriptome analysis, cytokine profiling, and protein analysis in patients' primary cells.

**Results:** We identified biallelic variants in close proximity to splice sites (c.1197G>C and c.1737+3A>G) in the *RNF31* gene. RNA extracted from patient cells showed alternatively spliced transcripts not present in control cells. Protein expression of HOIP and LUBAC was reduced in primary cells as shown by western blotting. Patient-derived fibroblasts demonstrated attenuated IL-6 production, while PBMCs showed higher TNF production after stimulation with proinflammatory cytokines. RNA sequencing of whole blood RNA and PBMCs demonstrated a marked transcriptome wide change including differential expression of type I interferon regulated genes.

**Conclusion:** We report the second case of HOIP deficiency with novel compound heterozygous mutations in *RNF31* and distinct clinical and molecular features. Our results expand on the clinical spectrum of HOIP deficiency and molecular signatures associated with LUBAC deficiency.

## Introduction

The signaling activity of nuclear factor kappa B (NF-κB) pathway is tightly regulated by post-transcriptional modifications including ubiquitination. Linear and Lys (K) 63 ubiquitination generally promote signaling activity, whereas K48 Ub chains act as a cellular protein degradation signal. The linear-ubiquitin assembly complex (LUBAC), consisting of HOIL-1-interacting protein (HOIP), Heme-oxidized IRP2 ubiquitin ligase-1 (HOIL-1) and SHANK-associated RH domain interactor (SHARPIN), specifically conjugates linear (Met1) ubiquitin chains to various target proteins in the canonical NF-κB pathway (1). LUBAC activity is counter-regulated by the linear ubiquitin specific deubiquitinase OTULIN. Defects in linear and K63 ubiquitination and deubiquitination processes result in immune dysregulations in mice and humans (2).

Patients with defects in the LUBAC components develop immunodeficiency, autoinflammation, and amylopectinosis of cardiac and/or skeletal muscle (Table S1) (3, 4). The previously reported HOIP deficiency is also noted to have lymphangiectasia leading to the malabsorption in the gastrointestinal tract, hypoalbuminemia, and systemic edema. Molecular investigations demonstrated that patients' fibroblasts and B cells were unresponsive to immune stimuli and failed to upregulate NF-κB activity consistent with the patient's observed immunodeficient phenotype. In contrast to immune responses in fibroblasts, peripheral blood mononuclear cells (PBMCs) of HOIP and HOIL1-deficient patients were highly responsive to IL-1 stimulation and produced proinflammatory cytokines IL-6 and MIP-1α.

Here we describe a second case of HOIP deficiency with compound heterozygous mutations in *RNF31*, who was clinically diagnosed with common variable immunodeficiency (CVID). Similar to the first case with HOIP deficiency, the patient presented with systemic inflammatory features but without evidence of amylopectinosis or lymphangiectasia. We also expand our understanding of the clinical manifestation of LUBAC deficiency using transcriptome analysis.

## Methods

### Targeted Next Generation Sequencing

Capture-based targeted next generation sequencing of 352 genes related to primary immunodeficiency and autoinflammation was conducted as described previously (5). Briefly, capture of the target regions, including the coding exons plus 50 flanking bases of 352 genes was performed with reagents from a custom designed HaloPlex Target Enrichment kit (Agilent Technologies), according to the HaloPlex Target Enrichment System Protocol. Samples were sequenced on the Ion Proton sequencer using the Ion PI chip v3 (Life Technologies). Read mapping and variant calling were performed using the Ion Torrent Suite software v4.4.2, against the UCSC hg19 reference genome. Variants were annotated using ANNOVAR (http://annovar.openbioinformatics.org/) (6).

### Sanger Sequencing

Coding regions as well as flanking intronic regions of the *RNF31* gene (RefSeq: NM_017999.4) were amplified by AmpliTaq Gold Fast PCR Master Mix (Thermo Fisher Scientific) and sequenced on 3130xl Genetic Analyzer (Applied Biosystems). For the subcloning analysis, the genomic region spanning from intron 6 to intron 9 of *RNF31* was PCR amplified, subcloned using TOPO TA cloning kit (Invitrogen) and sequenced. The data were analyzed by Sequencher (Gene Codes).

### RT-PCR

Whole blood RNA was extracted using PAXgene Blood RNA Kit (Qiagen) and reverse transcribed by SuperScript III First-Strand Synthesis SuperMix (Invitrogen). cDNA sequences of *RNF31* from exons 6 to 10 was PCR amplified, subcloned and Sanger sequenced.

### Intracellular Cytokine Staining

PBMCs were cultured with IL-1β (R/D, 10 ng/ml) and Monensin for 3, 6, and 24 h, were immunelabeled with antibodies against CD14 (BD; 557154), TNF (BD; 554513), IL-6 (BD; 561441), and IL-1β (Abcam; 16168) and with Yellow Live/Dead (Thermo; L34967), and were analyzed by CytoFLEX (BD).

### Lymphocyte Activation Assay

PBMCs were incubated with CellTrace violet (1 μM, Cell Proliferation Kit, Thermo), stimulated with anti-IgM (10 μg/ml, Jackson Lab; 109-006-129), CD40L (100 ng/ml, Enzo; ALX522-110-C010), IL-4 (10 ng/ml, Peprotech; 200-04), IL-21 (100 ng/ml, Peprotech; 200-21), CpG (1μM, Enzo; ALX746-006-C100), BAFF (100 ng/ml, Enzo ALX-522-025-C010), anti-CD3 (1 μg/ml, eBioscience; 16-0037-85), anti-CD28 (1 μg/ml, eBioscience; 16-0289-85) and PHA (1 μg/ml, Sigma; L9017). Seventy-two hours later the cells were stained with surface markers and analyzed by flow cytometer. Staining was performed with following antibodies from BD: CD3 (UCHT1), CD4 (RPAT4), CD8 (RPAT8), CD19 (HIB19) and CD80 (L307.4). For p-STAT1 detection, staining was performed using following antibodies from BD: CD4 (RPAT4), CD8 (SK1) and p-STAT1 (4a), and from Biolegend: CD3 (UCHT1), CD14 (M5E2) and CD45RA (HI100).

### Immunoblotting

Whole-cell lysates were prepared using ice-cold M-PER (Thermo Fisher Scientific) supplemented with protease inhibitor (Roche) and phosphatase inhibitor (Roche). Proteins were separated with Novex Tris-Glycine Gel Systems (Invitrogen) and transferred to PVDF membranes with iBlot2 systems (Invitrogen). After incubation with antibodies indicated below, proteins were visualized using ECL Plus Western blotting substrate (Thermo Fisher). Following antibodies were used for immunoblotting: HOIP (Abcam; ab46322), HOIL-1 (Sigma; MABC576), SHARPIN (CST; 12541), β –Actin (Santa Cruz; sc-47778), IkBα (CST; 4814), p-IkBα (CST; 2859), p-IKKα/β (CST; 2697), IKKα (CST; 11930), and TRIM25 (BD; 610570). Control primary fibroblast from an adult donor was purchased from ATCC (CS-201-012).

### LUBAC Reconstitution and Immunoprecipitation

*RNF31* ORF was subcloned into MSCV-N-Flag-HA-IRES-PURO vector (Addgene; 41033) using Gateway system (Invitrogen). The two mutant *RNF31* plasmids lacking exon 7 (c.810-1197) and exon 9 (c.1489-1737) were constructed by site-directed mutagenesis. SHARPIN (#50014) and HOIL-1 (#50016) expressing plasmids were purchased from Addgene. These plasmids were transiently expressed in HEK293T cells using Lipofectamine 2000 (Invitrogen). After 48 h cells were lysed using lysis buffer containing 30 mM Tris-HCl, 150 mM NaCl, 1% Triton-X 100, 1% protease inhibitor mixture and phosphatase inhibitor (Roche). For immunoprecipitations, 500 μg of lysates were incubated with anti HA affinity gel (Sigma; E6779) overnight at 4°C, washed five times with lysis buffer and subjected to immunoblotting.

### RNA Sequencing

Total RNA was isolated from whole blood collected in PAXgene Blood RNA Tubes using PAXgene Blood RNA Kit (PreAnalytiX) per manufacturer's instructions, and PBMCs using TRIzol (Thermo Fisher Scientific). Total RNA with high quality (RIN > 8) was used for cDNA library preparation using the TruSeq Stranded mRNA Library Preparation kit for NeoPrep (Illumina). Sequencing was performed on an Illumina HiSeq 3000 System in a 1 × 50 bp single read mode. Sequenced reads were mapped against the human reference genome (GRCh37) using TopHat v2.1.1 (7). Reads mapped to hemoglobin genes were removed from further analysis. Mapped reads were quantified using HTSeq (8). All the count data were normalized using TCC (9) and differentially expressed genes were detected using edgeR (10). Pathway enrichment analysis was performed using Ingenuity Pathway Analysis (IPA) software (Qiagen), respectively. The original RNAseq data is uploaded and available online (Gene Expression Omnibus: GSE118901).

### Statistics

All the statistical analyses were performed using R version 3.3.3 (R Foundation for Statistical Computing).

### Patient

**Figure 1:**
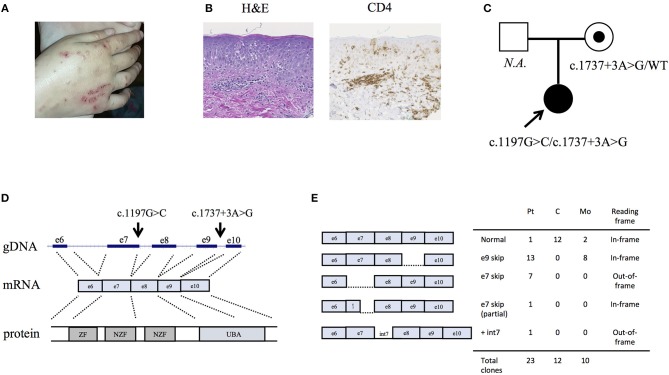
Clinical characteristics of the HOIP deficient patient. **(A)** Eczematous dermatitis. **(B)** Histopathology of the skin biopsy sample. **(C)** Information about the pedigree and the detected variants. **(D)** Genomic architecture of the *RNF31* gene through exons 6 to 10. ZF: zinc finger; NZF: Npl4-zinc finger; UBA: ubiquitin-associated. **(E)** Subcloning of the RT-PCR product demonstrated multiple forms of aberrant splicing. The table demonstrates the number of clones.

We studied an 8-year-old girl of mixed American ancestry born to non-consanguineous parents. At 7 months old she was diagnosed with polyarticular juvenile idiopathic arthritis after developing hip swelling and knee contracture. She was treated with corticosteroids, methotrexate, and eventually improved on TNF blockade with etanercept. At age 3, she developed recurrent fevers and suffered from severe bacterial, viral and fungal infections even after discontinuation of immunosuppressants (Table S1). Although she did not demonstrate hypogammaglobulinemia (IgG 645, IgM 25, and IgA 965 mg/dl), she lacked response to pneumococcal antigens upon vaccination. Given her clinical history, she was diagnosed with common variable immune deficiency (CVID). She also had a history of borderline cognitive delay and absence seizures, which might be attributed to a duplication on the chromosome 15q13.3 inherited from her asymptomatic mother (11). On examination at the age of 7 years old she was noted to have eczematous dermatitis (Figure 1A), splenomegaly, and clubbing of her toes and fingers. Skin biopsy demonstrated superficial perivascular chronic inflammation with dense infiltration of CD4^+^ and focal MPO^+^ cells (Figure 1B and Figure S1). Upper gastrointestinal endoscopy did not reveal histological evidence of lymphangiectasia. Serum creatine kinase, aldolase and echocardiography were normal, and muscle biopsy was not performed due to lack of clinical symptoms. Immunophenotyping of leukocyte surface markers demonstrated low memory B cells (Table S2). Consistent with the previous report of HOIP deficiency, the patient's B cell proliferation and CD80 expression were impaired after CD40 ligand (CD40L) stimulation and preserved after B cell receptor stimulation (Figures S2A,B). T lymphocyte proliferation following various stimuli were normal (Figure S3). Currently, the patient is stable with minimal inflammation on subcutaneous immunoglobulin supplementation.

## Results

**Figure 2:**
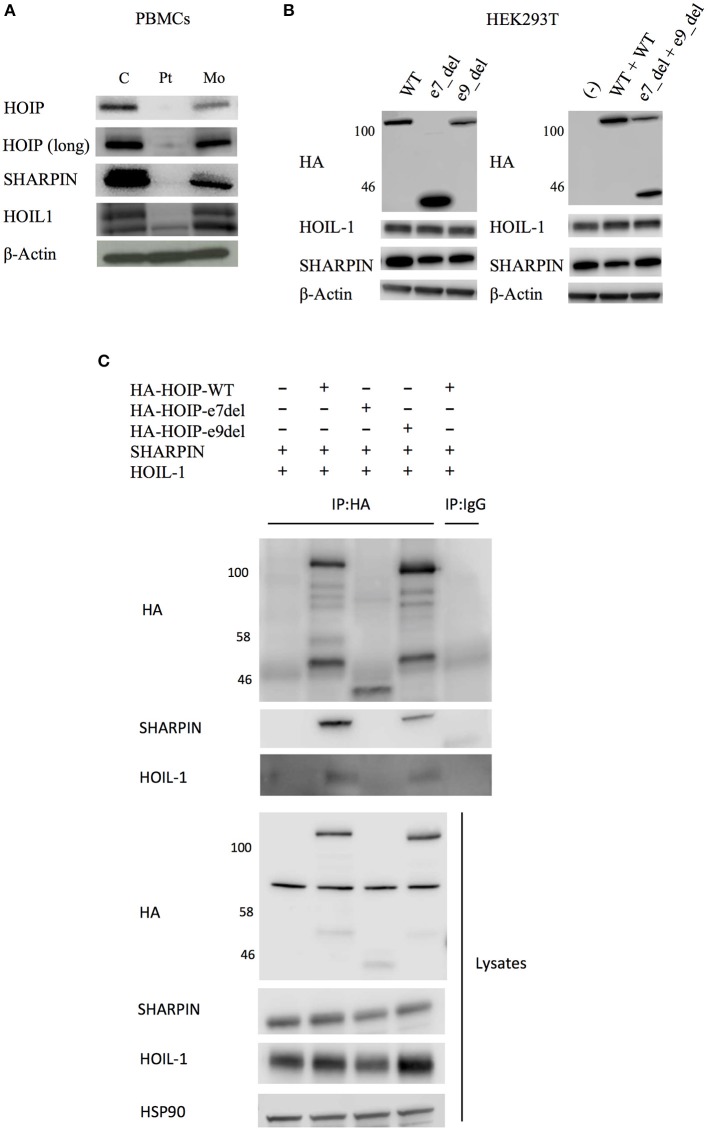
Effects of the identified *RNF31* mutations on LUBAC assembly. **(A)** The expression of LUBAC subunits in PBMCs. HOIP (long) indicates long exposure. C, unrelated age-matched control; Pt, the HOIP deficient patient; Mo, the mother. **(B)** Overexpression of HA-tagged mutant HOIP plasmids with SHARPIN and HOIL-1 in HEK293T cells. To mimic compound heterozygosity, two mutant plasmids were co-transfected. e7_del and e9_del indicate skipping of exon 7 and exon 9, respectively. **(C)** Immunoprecipitation of HA-tagged HOIP to assess the LUBAC assembly, in the presence of SHARPIN and HOIL-1 overexpression.

By targeted next generation sequencing of 352 immune-related genes we identified two single nucleotide variants in *RNF31*, which encodes HOIP: c.1197G>C; p.Q399H and c.1737+3A>G (Figure 1C and Table 1). The mother was heterozygous for the latter variant while her paternal sample was not available. We demonstrated that these variants are inherited *in trans* by sub-cloning of the patient's genomic DNA (Figure S4). We showed that the patient's RNA is aberrantly spliced and lacking exon 7 (c.810-1197) or exon 9 (c.1489-1737) (Figures 1D,E), while her mother's RNA was alternatively spliced and lacking only exon 9. The protein expression of HOIP, as well as HOIL-1 and SHARPIN, were markedly diminished in the patient's PBMCs, suggesting the destabilization of the LUBAC (Figure 2A). The exon 7 skipped transcript is out-of-frame and is translated into 39 kDa truncated protein, while the exon 9 skipped transcript is in-frame and lacks 84 amino acids (p.Arg496 to p.Lys579). To test the stability of these mutant proteins, we overexpressed individual *RNF31* mutant transcripts in HEK293T cells and co-expressed them together to mimic compound heterozygosity. The protein levels of these mutants were not noticeably reduced after overexpression, suggesting that instability of truncations in patient cells is likely bypassed by overexpression (Figure 2B).

**Table 1:** *RNF31* mutations identified in the patient.

**Chromosome**	**Genomic alteration^**a**^**	**cDNA alteration^**b**^**	**Amino acid alteration**	**Exon/ intron**	**ExAC allele frequency**
14	g.24619657G>C	c.1197G>C	p.Q399H	Exon 7	0
14	g.24620591A>G	c.1737+3A>G	-	Intron 9	0

a *Genome reference: GRCh37*.

b *cDNA reference: NM_017999.4*.

HOIP binds to SHARPIN and HOIL-1 via its Ubiquitin-Associated (UBA) domain (p.Arg480-p.Arg623), and these interactions are indispensable for the stabilization of LUBAC. Therefore, we next tested by immunoprecipitation whether these interactions are affected. As expected, the exon 7 deleted HOIP lacking the entire UBA domain had no interaction with SHARPIN and HOIL-1 (Figure 2C). Interestingly, although the interaction of exon 9 deleted HOIP with HOIL-1 was unchanged, its interaction with SHARPIN was markedly diminished. Recent structural analyses of LUBAC suggested that SHARPIN and HOIL-1 bind to two different modules within the UBA domain of HOIP (UBA-1: p.Arg480-p.Gln542 and UBA-2: p.Leu565-p.Arg623, respectively) (12, 13). The exon 9 mutant protein (lacking p.Arg496 to p.Lys579) lacks a large portion of the UBA-1 domain of HOIP that is required for the interaction with SHARPIN, and the loss of this interaction could cause destabilization of the endogenous LUBAC complex. Thus, our *in vitro* results indicate that both mutations affect the stability of LUBAC.

**Figure 3:**
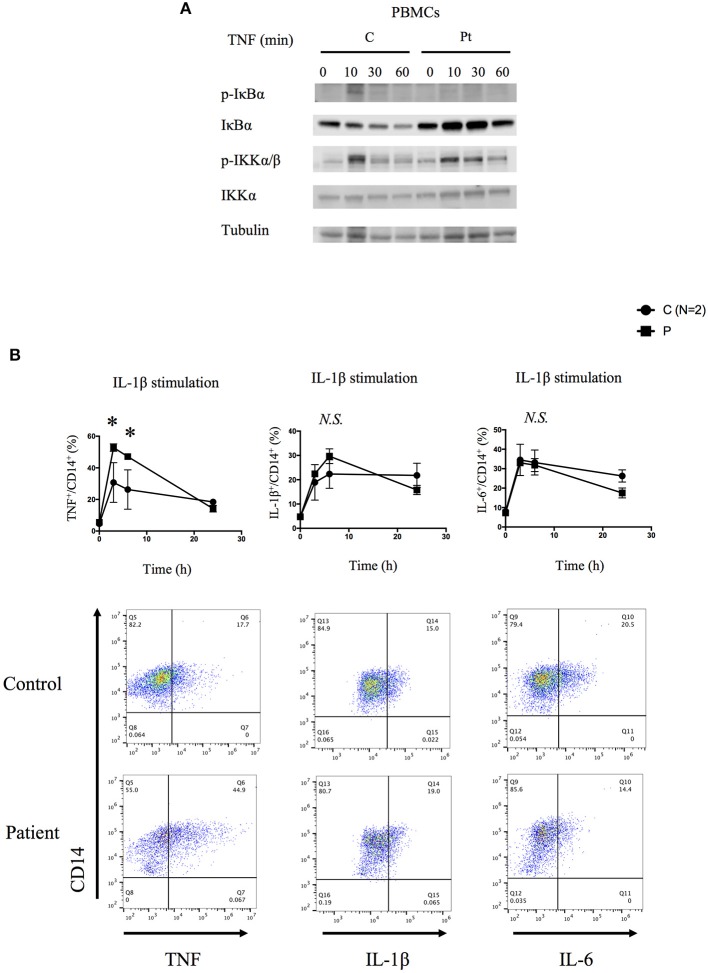
Molecular consequences of the HOIP deficiency. **(A)** PBMCs from the HOIP deficient patient showed decreased levels of phosphorylated IκBα and delayed phosphorylation of IKKα/β after TNF stimulation (20 ng/ml) compared with an unrelated healthy control. **(B)** Cytokine responsiveness of CD14^+^ monocyte subsets in the HOIP deficient patient and unrelated healthy controls (*N* = 2). Representative FACS plots are shown. Analysis was performed in triplicates. Two-way factorial ANOVA with Bonferroni adjustment was performed for the statistical analysis. ^*^adjusted *p* < 0.05.

We next tested the signaling activity of NF-κB by stimulating patient's PBMCs using TNF. Consistent with the immunodeficient phenotype and prior reports, these cells displayed reduced phosphorylation and decreased degradation of I-kappa-B alpha (IκBα), and delayed phosphorylation of IκB kinase α/β (IKKα/β) suggesting impaired activation of NF-κB (Figure 3A). Stimulated patient's CD14^+^ monocytes displayed increased accumulation of intracellular TNF, but not IL-1β or IL-6, in contrast to lower responsiveness of the patient's fibroblasts (Figure 3B and Figure S5). Serum cytokine measurement showed significantly increased levels of IL-6 at multiple time points, which is consistent with previous reports (Figure S6).

**Figure 4:**
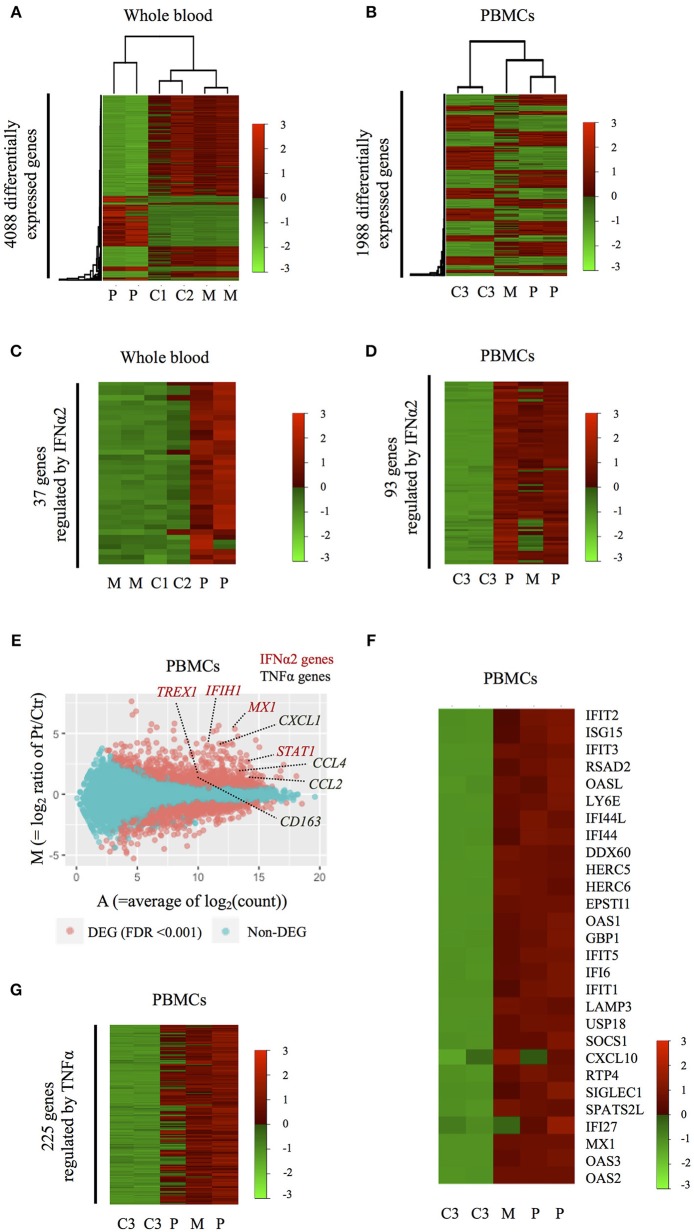
Inflammatory signatures of the HOIP deficient patient. **(A,B)** Heatmaps showing genome-wide expression change in the patient's whole blood **(A)** and PBMCs **(B)** obtained during symptom-free periods. C1-3, M, and P indicate healthy unrelated controls, the mother and the patient, respectively. **(C,D)** Heatmaps showing the expression patterns of differentially expressed genes regulated by IFNα2 in whole blood **(C)** and PBMCs **(D)**. **(E)** M-A plot analysis showing differentially expressed genes between PBMCs of the HOIP deficient patient and unrelated controls. Red dots indicate genes differentially expressed with statistical significance (edgeR, *p* < 0.001). **(F)** Heatmap showing the expression of 28 genes in PBMCs that are known to be upregulated in monogenic type I interferonopathies. **(G)** Heatmap showing the expression pattern of differentially expressed genes regulated by TNF.

We then studied the genome-wide impact of HOIP deficiency by RNA sequencing of the patient's unstimulated whole blood sample and isolated PBMCs collected during asymptomatic periods. Transcriptome-wide clustering demonstrated a unique profile for the proband compared to unrelated age-matched healthy controls (Figures 4A,B and Figure S7A). Pathway analysis demonstrated enrichment of interferon (IFN) α2-regulated genes exemplified by *MX1, TREX1, IFIH1, IRF7, IRF9*, and *STAT1* (Figures 4C–E, Figure S7B, and Table S3). The type I IFN signature, as defined by the set of 28 genes upregulated in monogenic interferonopathies (14), was enriched in the patient's PBMCs (Figure 4F), and less remarkable in the whole blood (Figure S7C). Additional pathway analysis of the RNAseq data identified a prominent TNF gene expression signature in the patient's PBMCs exemplified by *CCL2, CCL4, CXCL1*, and *CD163* (Figures 4E,G). Overall, the interferon-response gene cluster was ranked higher than the cluster of TNF-induced genes in PBMCs (Table S3). Type I IFN and TNF-mediated inflammatory transcriptome signatures were also identified in the PBMCs of the asymptomatic mother (Figures 4D,F,G and Figure S7A), which suggests a subclinical impact of HOIP haploinsufficiency in mononuclear hematopoietic cells.

**Figure 5:**
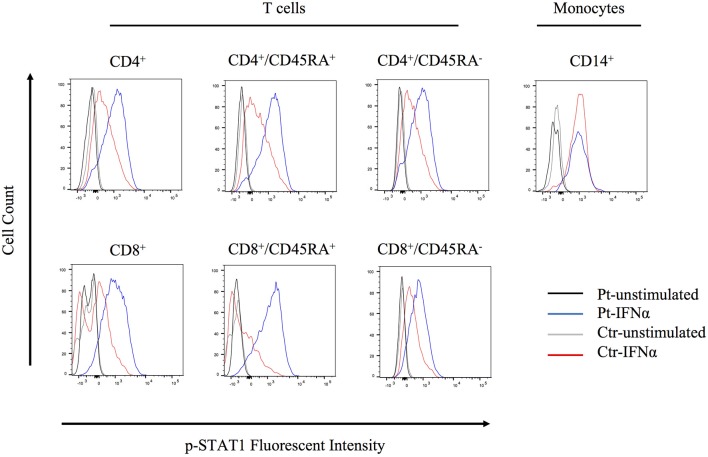
Flow cytometry analysis of STAT1 phosphorylation. PBMCs were stimulated with IFNα2 (20 ng/ml) for 20 min and CD4^+^, CD8^+^, and CD14^+^ cells were assessed for STAT1 phosphorylation.

The molecular pathophysiology of autoinflammation in LUBAC deficient patients is still unclear, in particular with regard to the interferon pathway. Inn et al previously reported that the LUBAC plays a role in regulating antiviral responses via degradation of TRIM25, a positive regulator of type I IFN production (15). Specifically, the baseline expression of TRIM25 protein is shown to be upregulated in HOIL-1 knock-out and HOIP knock-down mouse embryonic fibroblasts (MEFs) (15). However, we did not observe increased expression of TRIM25 in unstimulated HOIP deficient patient's PBMCs, compared to control cells (Figure S8). To investigate potential lineage-specific type I IFN signaling we stimulated patient's PBMCs with IFNα and examined T cell subsets and monocytes. We observed that the patient's T cells, especially CD4^+^/CD45RA^+^ and CD8^+^/CD45RA^+^ naïve subsets, had higher levels of phosphorylated STAT1 compared to healthy control cells (Figure 5). Interestingly, this induction was not observed in the patient's CD14^+^ monocytes, suggesting a lineage-specific role of LUBAC in T cells.

## Discussion

We herein describe the second case of HOIP deficiency manifesting with CVID, autoinflammation but without evidence for lymphangiectasia or amylopectinosis. The two cases of HOIP deficiency shared similar clinical features of immune dysregulations. They both suffered from severe bacterial and viral infections due to low number of memory B cells and impaired antibody production. The first HOIP deficient patient was noted to have severe T cell defects, such as T cell lymphopenia especially affecting naïve T population and impaired response to T cell stimulations, consistent with previous reports of HOIP knockdown experiments in the Jurkat cells (16) and specific *Rnf31* knock-in mice lacking the C-terminal half of the HOIP protein (17). However, these features were not observed in our patient, who showed normal T cell counts and normal response to T cell receptor-mediated stimulations, which might be attributed to her different genotype, her age, or other environmental factors. The first reported homozygous missense mutation p.Leu72Pro in the PUB domain of HOIP was also shown to impair the HOIP expression and stability of the LUBAC complex.

LUBAC plays a crucial role in regulation of innate and adaptive immune responses. LUBAC has been shown to maintain the stability of many receptor signaling complexes (RSCs) including TNFR1, TLRs, IL-1R, CD40, RIG-I like receptors and inflammasomes. These receptors are upstream of NF-kB pathway, which governs immune responses, cell cycle progression and cell death. LUBAC depletion leads to attenuation of NF-kB and the mitogen-activated protein kinases (MAPK) mediated signaling and increases cell death. Previous reports of patients with LUBAC deficiency demonstrated impaired NF-κB activation in mutant fibroblasts, while IL-1β stimulated patients' CD14^+^ monocytes had higher intracellular production of IL-6 and IL-1β. These lineage- specific signaling findings had been proposed to explain a complex immune phenotype of immunodeficiency and autoinflammation in LUBAC deficient patients (3, 4). Our data support these prior results, although we observed increased expression of TNF in IL-1β stimulated monocytes. High levels of IL-6 were found in the patient's serum samples while a significant upregulation of the type I IFN-induced transcripts was detected in PBMCs-derived RNA of the patient. These findings suggest that hypomorphic mutations in LUBAC could lead to hypersensitivity of myeloid cells resulting in increased production of many proinflammatory cytokines. Furthermore, we detected enhanced p-STAT1 signaling in T cells but not in monocytes. Thus, the type I interferon signature in PBMCs might be predominantly driven by T cells. Consistent with this finding, our patient has a history of severe eczematous skin rashes and her skin biopsy demonstrated a prominent dermal and perivascular infiltration of CD4^+^ T cells. Type I interferonopathy is a clinical entity in which enhanced type I IFN signaling activity plays a central role in its pathophysiology (18). At this point there is no strong evidence to categorize the HOIP deficiency as a new type I interferonopathy, and it is unclear whether these patients would benefit from treatment with JAK inhibitors. Our patient is not currently treated with any cytokine inhibitors and she remains stable solely on immunoglobulin replacement therapy. Studies of LUBAC deficient mice have suggested that mutant cells are susceptible to cell death (apoptosis and/or necroptosis) and this cellular defect has been shown to contribute to an inflammatory phenotype (19–23). The dysregulation in cell death pathway has been reported in human Mendelian diseases characterized by attenuated NF-κB signaling, such as RIPK1 deficiency and RELA haploinsufficiency (24–26). Therefore, the autoinflammatory phenotype in patients with HOIP and HOIL-1 deficiencies could be also attributed to the aberrant activation of programmed cell death. Although the source of autoinflammation in HOIP and HOIL1 deficiencies are still not well-understood, dysregulated LUBAC activity is expected to have cell-specific effects on many cellular pathways and functions.

We did not observe amylopectinosis in our patient, although this was not directly assessed using histopathology due to a lack of myopathic manifestations. In addition to the previous four cases of LUBAC deficiencies with immune dysregulation, two recent reports further described 14 cases of HOIL1 deficient cases associated with myopathy/cardiomyopathy but without immune phenotypes (27, 28), indicating that LUBAC deficient patients have a spectrum of clinical manifestations. The pathophysiology of amylopectinosis in the LUBAC deficiency is still unclear. Of note, loss-of-function mutations in the *EPM2B* gene that encodes an E3-ubiquitin ligase are reported to cause progressive epilepsy due to polyglucosan deposition in neuronal tissues (29), which may indicate a common role of the ubiquitin ligases in the regulation of glycogen metabolism. The clinical and histological absence of lymphangiectasia in our patient is noteworthy, because the vessel formation abnormality has been observed in the previous HOIP-deficient patient as well as in *Rnf31*-knock out mice (19). Possible explanations for this phenotypic difference include low level residual expression of HOIP in our patient (Figure 1E and Figure 2A), earlier age at evaluation, or other confounding genetic causes in the first report due to consanguinity (3).

Our study of a second HOIP-deficient patient helps both to confirm original clinical findings and mouse models and expands our knowledge about the clinical manifestations and mechanism of LUBAC dependent disease. We have specifically identified different inflammatory signatures in PBMCs that also bears evidence for a subclinical phenotype in mutation carriers. Finally, our case report emphasizes the spectrum of clinical features of HOIP deficiency in children and expands the differential diagnosis for CVID and systemic inflammation.

## Data Availability

The datasets generated for this study can be found in Gene expression omnibus, GSE118901.

## Ethics Statement

All subjects gave written informed consent, including consent to publish, in accordance with the Declaration of Helsinki. The protocol was approved by the NHGRI Institutional Review Board.

## Author Contributions

HO, DB, DK, and IA designed the project and wrote the manuscript. HO, PH, MI, SR, and LN were involved in collecting phenotypic data from the patient. HO, DB, HK, NS, WT, MG, and SR performed or analyzed experiments. JS and GG-C performed next generation sequencing.

### Conflict of Interest Statement

The authors declare that the research was conducted in the absence of any commercial or financial relationships that could be construed as a potential conflict of interest.

## Supplementary Material

Figure S1 Histological examination of the HOIP deficient patient demonstrated dermatitis, associated with global perivascular infiltration of CD3^+^ and CD4^+^ T cells and focal MPO^+^ neutrophils.

Figure S2 B lymphocytes (CD3^−^CD19^+^) from the HOIP deficient patient showed decreased response to CD40 ligand stimulation. **(A)** PBMCs were stained with CellTrace Violet, cultured with various stimuli for 72 h and analyzed by flow cytometer. **(B)** PBMCs were cultured with various stimuli for 72 h, and the expression levels of CD80 were analyzed by flow cytometer. MFI: mean fluorescence intensity. Red: the HOIP deficient patient, blue: unrelated age-matched healthy control.

Figure S3 Normal T lymphocyte proliferation in the HOIP deficient patient. PBMCs were stained with CellTrace Violet, cultured with anti-CD3, anti-CD3/CD28, or phytohemagglutinin (PHA) for 72 h, and analyzed by flow cytometer. Gray: unstimulated, red: the HOIP deficient patient, blue: unrelated healthy control.

Figure S4 Subcloning of the patient's genomic DNA demonstrated that the *RNF31* variants are inherited *in trans*.

Figure S5 Cytokine responsiveness of dermal fibroblasts from the HOIP deficient patient. Analysis was performed in triplicates. Representative FACS data are shown below. Two-way factorial ANOVA with Bonferroni adjustment was performed for the statistical analysis. ^*^adjusted *p* < 0.05.

Figure S6 Serum IL-6 analysis of the patient and unrelated healthy controls (*N* = 8). The patient's samples were taken when the patient was asymptomatic and not placed on immunosuppressants. The ELISA measurement was performed in technical duplicates. Mann-Whitney U test was performed for the statistical analysis. ^***^*p* < 0.001.

Figure S7 Transcriptomic features of the HOIP deficient patient. **(A)** Hierarchical clustering of the RNAseq of whole blood and PBMCs of the HOIP deficient patient. **(B)** M-A plot analysis showing differentially expressed genes in whole blood RNAseq of the HOIP deficient patient and the healthy controls. Red dots indicate gene expression levels with statistical significance (edgeR, *p* < 0.001). **(C)** Heatmap showing the expression of 28 genes in whole blood RNA that are known to be upregulated in monogenic type I interferonopathies.

Figure S8 Expression levels of TRIM25 in unstimulated PBMCs of the HOIP deficient patient. HeLa cell extract was used as a positive control for the western blot. C, unrelated healthy control; P, HOIP deficient patient.

Table S1 Phenotypic comparison of the LUBAC deficiency patients with immune dysregulations.

Table S2 Lymphocyte surface markers.

Table S3 Upstream pathway terms enriched in the HOIP deficient patient's PBMCs.
